# [Retracted] Esomeprazole affects the proliferation, metastasis, apoptosis and chemosensitivity of gastric cancer cells by regulating lncRNA/circRNA-miRNA-mRNA ceRNA networks

**DOI:** 10.3892/ol.2026.15793

**Published:** 2026-08-03

**Authors:** Qian Xu, Xiyun Jia, Qian Wu, Lei Shi, Zihan Ma, Nan Ba, Han Zhao, Xingzhou Xia, Zisen Zhang

Oncol Lett 20: 329, 2020; DOI: 10.3892/ol.2020.12193

Following the publication of the above paper, it was drawn to the Editor's attention by a concerned reader that, regarding the flow cytometric plots shown in Fig. 4A on p. 5, the ‘Control’ and ‘ESO’ pair of panels, the ‘DDP’ and ‘DDP+EDO’ pair of panels, and the ‘ADM / ADM+ESO / ADM+DDP / ADM+DDP+ESO’ panels grouped together, separately appeared to show similar groupings of dots, which would not have been anticipated if these experiments had been performed discretely under different experimental conditions, suggesting a fundamental flaw either in the way in which these experiments were performed, or in how the results were outputted.

The Editor of *Oncology Letters* has decided that this paper should be retracted from the Journal on account of a lack of confidence in the presented data. After contacting the authors, they did not accept the decision to retract the paper. The Editor apologizes to the readership for any inconvenience caused.

